# The Longevity-Associated Variant of BPIFB4 Reduces Senescence in Glioma Cells and in Patients’ Lymphocytes Favoring Chemotherapy Efficacy

**DOI:** 10.3390/cells11020294

**Published:** 2022-01-15

**Authors:** Annibale Alessandro Puca, Valentina Lopardo, Francesco Montella, Paola Di Pietro, Daniela Cesselli, Irene Giulia Rolle, Michela Bulfoni, Veronica Di Sarno, Giorgio Iaconetta, Pietro Campiglia, Carmine Vecchione, Antonio Paolo Beltrami, Elena Ciaglia

**Affiliations:** 1 Department of Medicine, Surgery and Dentistry “Scuola Medica Salernitana”, University of Salerno, Via Salvatore Allende, Baronissi, 84081 Salerno, Italy; vlopardo@unisa.it (V.L.); fmontella@unisa.it (F.M.); pdipietro@unisa.it (P.D.P.); giaconetta@unisa.it (G.I.); cvecchione@unisa.it (C.V.); 2 Cardiovascular Research Unit, IRCCS MultiMedica, 20138 Milan, Italy; 3 Department of Medicine, University of Udine, 33100 Udine, Italy; daniela.cesselli@uniud.it (D.C.); irenegiulia.rolle@gmail.com (I.G.R.); michela.bulfoni@uniud.it (M.B.); antonio.beltrami@uniud.it (A.P.B.); 4 Department of Pharmacy, University of Salerno, Via G. Paolo II 132, Fisciano, 84084 Salerno, Italy; vdisarno@unisa.it (V.D.S.); pcampiglia@unisa.it (P.C.); 5 Department of Neurosurgery, University Hospital “San Giovanni di Dio e Ruggi d’Aragona”, 84131 Salerno, Italy; 6 Department of AngioCardioNeurology, IRCCS Neuromed, Pozzilli, 86077 Isernia, Italy

**Keywords:** senescence, glioma, longevity, PBMCs, chemotherapy

## Abstract

Glioblastoma (GBM) is the most common primary brain cancer with the median age at diagnosis around 64 years, thus pointing to aging as an important risk factor. Indeed, aging, by increasing the senescence burden, is configured as a negative prognostic factor for GBM stage. Furthermore, several anti-GBM therapies exist, such as temozolomide (TMZ) and etoposide (ETP), that unfortunately trigger senescence and the secretion of proinflammatory senescence-associated secretory phenotype (SASP) factors that are responsible for the improper burst of (i) tumorigenesis, (ii) cancer metastasis, (iii) immunosuppression, and (iv) tissue dysfunction. Thus, adjuvant therapies that limit senescence are urgently needed. The longevity-associated variant (LAV) of the bactericidal/permeability-increasing fold-containing family B member 4 (BPIFB4) gene previously demonstrated a modulatory activity in restoring age-related immune dysfunction and in balancing the low-grade inflammatory status of elderly people. Based on the above findings, we tested LAV-BPIFB4 senotherapeutic effects on senescent glioblastoma U87-MG cells and on T cells from GBM patients. We interrogated SA-β-gal and HLA-E senescence markers, SASP factors, and proliferation and apoptosis assays. The results highlighted a LAV-BPIFB4 remodeling of the senescent phenotype of GBM cells, enhancement of their sensitivity to temozolomide and a selective reduction of the T cells’ senescence from GBM patients. Overall, these findings candidate LAV-BPIFB4 as an adjuvant therapy for GBM.

## 1. Introduction

Glioblastoma (named multiforme for its heterogeneity; GBM) is currently recognized as the most aggressive malignant primary brain tumor [1].

Surgical resection followed by radiotherapy in combination with cycles of temozolomide (TMZ)-based therapy represent the standard-of-care treatments for GBM patients [2]. Presumably due to the complex heterogeneity of the GBM landscape, this canonical treatment approach dramatically fails since the recurrence rate is more than 90% [3]. In addition, emerging immunotherapeutic strategy (i.e., immune checkpoint blockers) seems to lack a durable response with no recurrence in GBM patients [4]. At the same time, in combination with etoposide (ETP), a topoisomerase II inhibitor, no relevant effect on postresection residual disease was documented [5,6]. Looking for the major determinants of the failure in anticancer drug response, senescence outcome has a critical role.

Senescence is thought to be a major barrier to tumor formation, as it limits the replicative potential of many tumors including melanoma [7], hepatocellular carcinoma [8], and medulloblastoma [9].

On the other hand, recent studies show that senescence is involved in oncogenesis and cancer aggressiveness, as senescent tumor cells can serve as a reservoir of secreted factors with mitogenic, antiapoptotic, and angiogenic activities [10]. Senescent tumor cells recruit CCR2-positive myeloid cells, enhancing hepatocellular carcinoma growth and worsening the prognosis of patients with hepatocellular carcinoma through NK cell inhibition [11]. Further, senescence of HER-2-positive breast cancer cells can promote cancer progression [12]. It has also been reported that cisplatin, by inducing senescent melanoma cells, improves the growth of non-senescent melanoma cell compartment [13]. Furthermore, therapy-triggered senescence may induce cancer stem-like cells [14] responsible for chemoresistance due to the senescence intrinsic activity of cellular reprogramming [15].

In glioblastoma, the above scenario (i.e., cellular senescence) has been reported in response to the combination of TMZ with radiotherapy, which is administered after surgery in GBM patients [16]. The senescence outcome avoids the proliferative capacity of cancer cells in a p53-dependent manner and by inducing CHK1/CDC25c-dependent G2/M arrest [16]. Similarly, ETP, by inducing DNA damage, interferes with DNA repair processes, thus triggering both apoptosis and stress-induced senescence [17]. Despite the promising therapeutic effect of temozolomide or etoposide, physiologically cancer senescent cells remain metabolically active, and this brings attention to the potential side effects of cancer cell senescence. Regarding this, cancer senescent cells, characterized by increased SA-β-gal activity [18], formation of senescence-associated heterochromatin foci [19], and induction of senescence-associated DNA damage [20], show a spectrum of pro-inflammatory chemokines and cytokines (referred to as senescence-associated secretory phenotype, SASP; [21]). The combination of these changes promotes tumor growth and evasion from therapy-induced senescence due to the fact of both autocrine and paracrine effects. These lead to proliferation, apoptosis resistance, and angiogenesis generating a self-sustained deleterious pro-inflammatory and immunosuppressive microenvironment (such as matrix metalloproteinases, IL-1, IL-6, IL-8, and TGF-β) [22]. Based on these premises, glioblastoma becomes profoundly resistant to temozolomide, inducing DNA repair mechanisms [23], activation of the multidrug resistance (MDR) system [24], proliferation of glioblastoma stem cells [25], and evasion from the immune surveillance. The immunosuppressive microenvironment of GBM inhibits T-cell proliferation and activation, downregulates MHC expression in microglia cells affecting their antigen-presenting ability, hyper stimulates the activity of Treg cells, and causes anergy (i.e., perpetually inactivation), exhaustion (i.e., hyporesponsive state), or senescence in infiltrating T cells [26,27].

Notably, age is a negative prognostic factor for GBM stage, and, with age, senescence steadily increases. As a consequence, senescence is reported as a shared hallmark of cancer and aging. Indeed, if on the one hand senescence is a mechanism protecting cells from tumorigenic transformation, on the other hand, it is also true that senescence marks the aging of the organism that becomes more inclined to cancer development [28].

As statistics have demonstrated, GBM patients are mainly elderly people given the median age of diagnosis of 64 years and an average survival time of approximately 15 months post-diagnosis [29,30]. Of note, the less efficient immune system in elderly (referred to as immunesenescence, [31]) may be another key factor in developing glioblastoma with worse prognosis.

The longevity-associated variant encoded by a four-SNP haplotype of BPIFB4 (i.e., bactericidal/permeability-increasing fold-containing family B member 4) gene was previously found to be able to enhance health/longevity and cellular homeostasis both in several (in vitro and murine) models of human diseases (COVID-19, Huntington’s disease, heart failure, atherosclerosis, diabetic complications, frailty, etc.) and in frail people [32,33,34,35,36]. High levels of the secreted BPIFB4 protein are found in serum of long-living individuals (LLIs) classifying their healthy status [37]. In these contexts, LAV-BPIFB4 demonstrates a regulatory role in the immune system’s remodeling/adaptation and the recovery of the inflammatory balance mirroring what occurs in LLIs. LAV-BPIFB4 regulates the macrophages pro-resolving M2-like polarization state (both in microglia and atherosclerotic plaque) and improves the inflammatory balance (i.e., reduces IL-1β and TNF-α levels and increases IL-33 levels in atherosclerotic process but also increases IL-1β and IL-18 levels to contrast SARS-CoV-2 infection) [38]. Definitively, LAV-BPIFB4 exerts a balancing action to finely tune the age-induced immunological dysfunction [39]. Whether LAV-BPIFB4 influences senescence burden in the cancer microenvironment and immune escaping of GBM is completely unknown. Thus, since LLIs are protected from age-related pathologies, their genetic risk factors for healthy longevity might be helpful for developing therapeutic approaches. On these bases, we firstly indagated the role of LAV-BPIFB4 in shaping the senescence phenotype and microenvironment in an in vitro model of glioblastoma (i.e., U87-MG cells). Subsequently, we evaluated a putative effect of LAV-BPIFB4 in lessening the senescent status of innate and adaptive immune cells from low- and high-grade GBM patients.

## 2. Methods

### 2.1. Cell Culture and Treatment

The human glioma cell line, U87-MG, was obtained from CLS Cell Lines Service GmbH (Eppelheim, Germany). Small pieces of brain tissue containing tumor were collected at the time of craniotomy for tumor resection at the Neurosurgery Service of “Santa Maria della Misericordia” Medical Hospital (Udine, Italy) and handled for further analysis. The tumors were diagnosed as glioma, GBM low grade (WHO grade II, *n* = 6) or glioblastoma multiforme, GBM high grade (WHO grade IV, n = 7). Peripheral blood mononuclear cells (PBMCs) from GBM patients or healthy controls were harvested from whole blood collected in hospital. All GBM samples were collected in accordance with the ethical standards of the Institutional Committee of “Santa Maria della Misericordia” Medical Hospital (Udine, Italy), protocol No. 196/2014/Em, 18 November 2014. The patients were informed about the use of their biological material for research purposes. The U87-MG cells were cultured in EMEM (Gibco^®^, Thermo Fisher Scientific, Waltham, MA, USA) supplemented with 10% heat-inactivated FBS (Gibco^®^, Thermo Fisher Scientific, Waltham, MA, USA), 1% L-glutamine (Aurogene, Rome, Italy), 1% antibiotic mixture (Aurogene, Rome, Italy), 1% sodium pyruvate (Aurogene, Rome, Italy), and 1% non-essential amino acids (MEM NEAA, Gibco^®^, Thermo Fisher Scientific, Waltham, MA, USA). All cell cultures were maintained at 37 °C in a humidified 5% CO_2_ atmosphere. The U87-MG cell line was treated with temozolomide (Sigma–Aldrich, Darmstadt, Germany) at 100 μM for 96 h in the presence or absence of recombinant LAV-BPIFB4 protein at 18 ng/mL. Additionally, in order to evaluate the effect of LAV-BPIFB4 on temozolomide-induced senescence and apoptosis, U87-MG cells were pre-treated with temozolomide (100 μM) for 24 h, the medium was discarded, and fresh medium was added for an additional 5 days in the presence or absence of LAV-BPIFB4 (18 ng/mL) for the last 48 h. Similarly, U87-MG cells were also treated with etoposide (6 μM) for 24 h. After 24 h, the medium was discarded, and fresh medium was added for an additional 5 days in the presence or absence of LAV-BPIFB4 (18 ng/mL) for the last 48 h.

The PBMCs were extracted from whole blood by Ficoll density gradient (Histopaque^®^-1077, Sigma–Aldrich, Darmstadt, Germany). After separation, the PBMCs were washed and collected in RPMI-free (Gibco^®^, Thermo Fisher Scientific, Waltham, MA, USA) or RPMI (Gibco^®^, Thermo Fisher Scientific, Waltham, MA, USA) supplemented with 10% (v/v) fetal serum bovine (FBS, Gibco^®^, Thermo Fisher Scientific, Waltham, MA, USA), 1% (v/v) penicillin–streptomycin (Aurogene, Rome, Italy), 1% (v/v) MEM non-essential amino acids (MEM NEAA, Gibco^®^, Thermo Fisher Scientific, Waltham, MA, USA), and 1% (v/v) sodium pyruvate (Aurogene, Rome, Italy) for the subsequent experiments. PBMCs were also treated with recombinant LAV-BPIFB4 (18 ng/mL) for 48 h to perform further FACS analysis.

### 2.2. Senescence Induction and Detection

Glioblastoma cells (U87-MG) were treated with etoposide (6 μM) to induce senescence. After 24 h from treatment, the medium containing etoposide was removed, fresh medium was added, and the cells were incubated at 37°C for another 5 days without further changes of medium culture. A cellular senescence detection kit, SPiDER-ßGal (Dojindo Laboratories, Rockville, MD, USA), was used following the producer’s protocol for FACS analysis to verify the senescence in the cells.

### 2.3. Cytofluorimetric Analysis

PBMCs from GBM patients and healthy controls were stained with mAb against human CD3 FITC (Miltenyi Biotec, Bergisch Gladbach, Germany), CD56 PerCP-Cy5.5 (BioLegend, San Diego, CA, USA), NKp44 APC (Miltenyi Biotec, Bergisch Gladbach, Germany), CD11b APC (BioLegend, San Diego, CA, USA), CD14 PerCP Cy5.5 (BioLegend, San Diego, CA, USA), and CD206 (BioLegend, San Diego, CA, USA), CD163 APC (Miltenyi Biotec). The U87-MG cells were stained with mAb against human MDR PerCP Cy5.5 (BioLegend, San Diego, CA, USA). After 30 min incubation at 4 °C in the dark, cells were washed, centrifuged, and resuspended in staining buffer for the FACS analysis. For each test, cells were analyzed using a FACSVerse flow cytometer (BD Biosciences, Swindon, UK). The U87-MG cells (treated as mentioned above), healthy controls, and patients’ PBMCs were also stained for Sa-β-galactosidase and incubated for 15 min at 37 °C in the dark without washing before FACS analysis.

### 2.4. Cytokine Detection

IL-8, IL-6, IL-1β, and MCP-1 levels in the conditioned media of senescent U87-MG cells and IL-1α, IL-6, IL-8, and IL-10 levels in the conditioned media of healthy controls and patients’ PBMCs were determined using a beads-based multiplex ELISA (LEGENDplexTM, BioLegend, San Diego, CA, USA). Medium was incubated for 2 h with the beads and for 1 h with the detection antibodies, followed by 30 min incubation with SA-PE. After washing, the beads were resuspended in washing buffer and acquired using a FACSVerse flow cytometer (BD Biosciences, Swindon, UK). Data were analyzed with the LEGENDplex Data Analysis Software.

### 2.5. Proliferation Assay

U87-MG cell proliferation was evaluated by measuring BrdU incorporation into DNA (BrdU colorimetric assay kit; Roche Applied Science, South San Francisco, CA, USA) following treatment with temozolomide (100 μM) combined with increasing doses of LAV-BPIFB4 (0–144 ng/mL) for 96 h or following treatment with LAV-BPIFB4 at 18 ng/mL without temozolomide. Newly synthesized BrdU-DNA was determined on an ELISA plate reader (Thermo Scientific, Waltham, MA, USA) at 450 nm. All experiments were performed in triplicate, and the relative cell growth was expressed as a percentage of the untreated control cells (set to 100%) to allow for an unwanted source of variation.

### 2.6. Cytotoxicity Assay

The Cell Counting Kit-8 (CCK-8 Cat. CK04, Dojindo Laboratories, Rockville, MD, USA) assay was performed on U87-MG cells after treatment with LAV-BPIFB4 (18 ng/mL) for 48–72–96 h. CCK-8 solution (10 μL) was added to the U87-MG cell suspension incubated for 1–4 h in a humidified incubator. Absorbance was detected at a wavelength of 450 nm.

### 2.7. Apoptosis Assay

Annexin V FITC Apoptosis Detection Kit (Cat. AD10, Dojindo Laboratories, Rockville, MD, USA) was employed to evaluate U87-MG cell apoptosis after treatment with temozolomide (100 μM) in the presence or absence of LAV-BPIFB4 (18 ng/mL). Then, 2.5 μL of Annexin V FITC Conjugate and 2.5 μL of propidium Iodide PE Solution were added to 50 μL of the cell suspension (50,000 cells/mL). After incubation for 15 min at room temperature with light protection, 200 μL of Annexin V Binding Solution was added for FACS analysis.

### 2.8. Western Blotting

The U87-MG cells were washed with PBS (Gibco^®^, Thermo Fisher Scientific, Waltham, MA, USA), harvested, and lysed in ice-cold RIPA lysis buffer (50 mM Tris-HCl; 150 mM NaCl; 0.5% Triton X-100; 0.5% deoxycholic acid; 10 mg/mL leupeptin: 2 mM phenylmethylsulphonyl fluoride; 10 mg/mL aprotinin, phosphatase inhibitor, and protease inhibitor). After centrifugation (13,000 rpm for 20 min at 4 °C) to remove cell debris, proteins were quantified. Approximately 25 μg of proteins were separated on 10% SDS-PAGE at 90 V for 1 h and at 120 V for 1 h and then transferred to a nitrocellulose membrane. After blocking with 5% nonfat dried milk powder (PanReac AppliChem, Darmstadt, Germany) in Tris-buffered saline containing 0.1% Tween-20 (TBST) for 1 h at room temperature, the membranes were incubated overnight with the following primary antibodies Abs: Ab anti-p16 (BioLegend #675602, mouse mAb 1:1000, San Diego, CA, USA), Ab anti-p21 (Santa Cruz Sc6246, mouse mAb 1:1000, Santa Cruz, CA, USA) Ab anti-β-actin (Abcam #49900, mouse mAb 1: 50,000, Cambridge, UK). Immunodetection of specific proteins was carried out with horseradish peroxidase-conjugated donkey anti-mouse IgG (Bio-Rad, California, USA), using the enhanced chemiluminescence (ECL) system (Thermo Fisher Scientific, Waltham, USA) according to the manufacturer’s instructions and then exposed to X-ray films (Thermo Fisher Scientific, Waltham, USA). Western blot data were analyzed using Photoshop software to determine the optical density (OD) of the bands. The OD readings of phosphorylated proteins were expressed as a ratio relative to the total protein and β-actin.

### 2.9. Statistical Analysis

Statistical analysis was performed for all of the experiments using GraphPad Prism 6.0 software for Windows (GraphPad software, San Diego, CA, USA). For each type of assay or phenotypic analysis, data obtained from multiple experiments were calculated as the mean ± SD and analyzed for statistical significance using ANOVA followed by Tukey’s correction for multiple comparisons.

## 3. Results

### 3.1. Recombinant Human LAV-BPIFB4 Influenced GBM Cell Senescence Rate

The ability of LAV-BPIFB4 to enhance health/longevity and cellular homeostasis led us to investigate its putative senotherapeutic action in GBM, a neoplasm of older adults in which senescence, the main driver of aging, also supports cancer progression and recurrence after surgery. To this end, we chose to analyze the phenotype of the senescent U87-MG glioma cell line in vitro in the presence or absence of recombinant human LAV-BPIFB4 (18 ng/mL). Further, according to the literature, we started to use a low dose (6 μM) of etoposide (ETP) to induce DNA damage as a conventional genotoxic stress, leading to cellular senescence [40].

**Figure 1:**
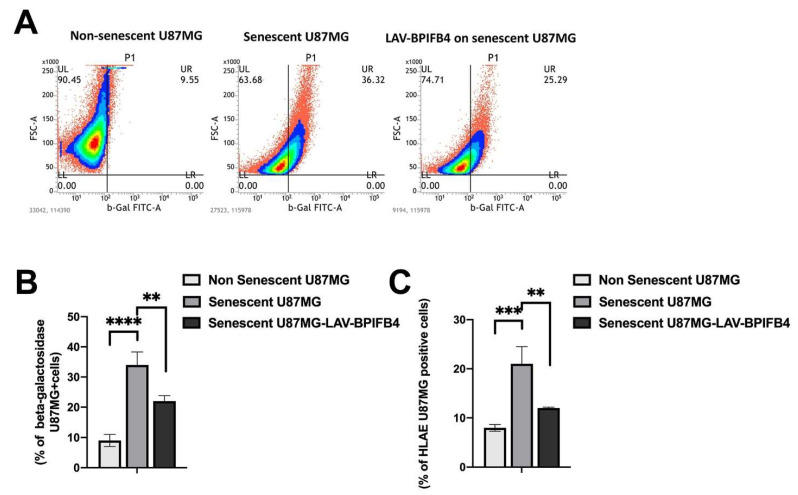
Senescence analysis in the U87-MG glioblastoma cell line. Cytofluorometric analysis of Spider-β galactosidase (spider-βGal), common senescence marker, in the U87-MG glioma cell line after 24 h of treatment with etoposide (6 μM) and 5 days resting in the presence or absence of 18 ng/mL of rhLAV-BPIFB4 for the last 48 h. The treatment with etoposide induced senescence as shown in (**A**) (middle graph); after the treatment with 18 ng/mL of rhLAV-BPIFB4 the spider-βGal values decreased (right graph in (**A**)). (**B**) Bar graph reporting the mean percentage values ± SD of β galactosidase viable cells from 3 independent experiments. (**C**) Cytofluorimetric analysis of the HLA-E expression on U87-MG cells’ surfaces in the control and in ETP-treated cells with or without rhLAV-BPIFB4 for the 5 days resting as indicated in Section 2. The bar graphs report the mean ± SD of the percentage of positive cells in the different conditions. Statistical analysis by two-way ANOVA with Tukey’s test for multiple comparison was conducted. (** *p* < 0.001, *** *p* < 0.0001, **** *p* < 0.00001).

As expected, when we exposed U87-MG to ETP for 5 days, we found that the chemotherapeutic agent significantly increased the rate of U87-MG senescence-associated β-galactosidase (SA-β-gal)-positive cells, a surrogate marker of senescence, as detected by flow cytometry analysis (Figure 1A). Of note, when U87-MG cells were treated with recombinant LAV-BPIFB4 during the last 48 h after ETP exposure, SA-β-gal-positive cell populations significantly decreased (Figure 1B). Further, as rhLAV-BPIFB4 neither affected the proliferative index nor the survival of the parental non-senescent U87-MG cells (Supplementary Materials Figure S1A–C), we can exclude LAV-BPIFB4 toxicity as a potential cause of the less accumulation of SA-β-gal in senescent cells.

The reduction of the senescence burden well correlated with the downregulation of HLA-E expression, which is commonly associated to the persistence of the senescent state (Figure 1C) [41]. Taken together, these first results indicate that LAV-BPIFB4 treatment might be able to blunt, in a specific manner, the senescence degree in GBM cells.

### 3.2. Recombinant Human LAV-BPIFB4 Decreased SASP in ETP-Treated Senescent GBM Cells

**Figure 2:**
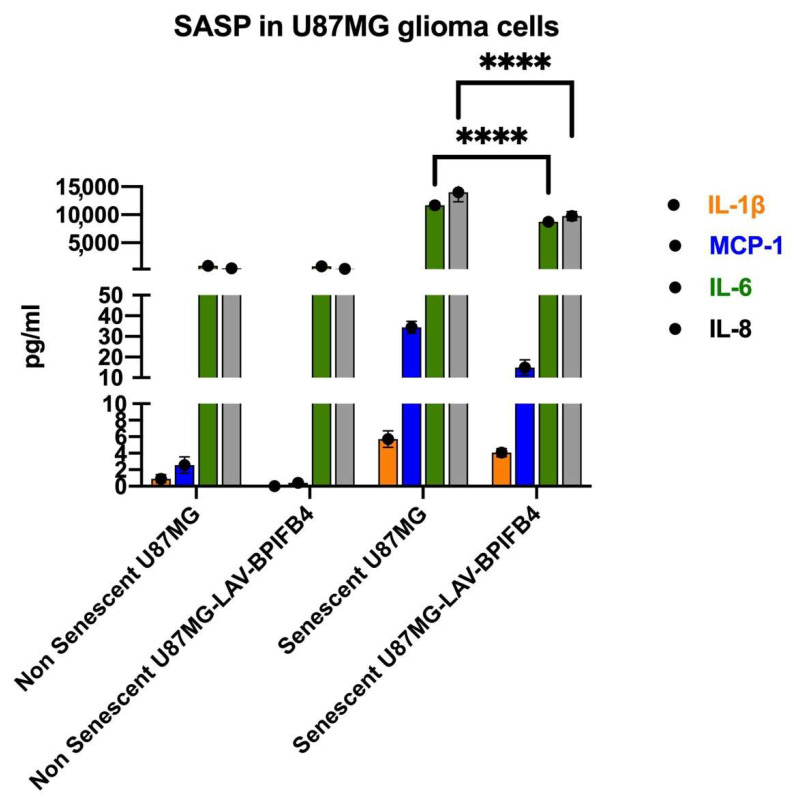
Cytokine analysis of Senescent U87-MG glioblastoma cell line. Secretory profile of U87-MG glioma cell line after 24 h treatment with etoposide (6 μM) and 5 days resting in the presence or absence of 18 ng/mL of rhLAV-BPIFB4 for the last 48 h as detected by multiplex ELISA of the cell medium. The etoposide induced the secretion of some SASP factors: IL-1β, MCP1, IL-6, and IL-8; the 48 h treatment with rhLAV-BPIFB4 modulated this secretory phenotype. Statistical analysis by two-way ANOVA with Tukey’s test for multiple comparison was conducted. (**** *p* < 0.0001).

As senescent cells may express the senescence-associated secretory phenotype (SASP) to induce and maintain the senescence phenotype in normal surrounding cells to properly burst tumor progression, we moved to test the senotherapeutic effect of LAV-BPIFB4 on the secretion of main SASP factors which included cytokines (IL-6, IL-8 and IL-1β) and other factors such as monocyte chemotactic protein 1 (MCP1). To this end we examined the protein levels of SASP in senescent GBM cells and in non-senescent counterpart for comparison by using multiplex ELISA. The results showed that the MCP1, IL-1β, IL-6, and IL-8 levels increased in ETP-treated U87-MG cells compared to non-senescent counterpart. The co-treatment with LAV-BPIFB4 for the last 48 h decreased all SASP factors by reaching statistical significance for IL-6 and IL-8 levels (Figure 2). No effects were reported for LAV-BPIFB4 on basal cyto-chemokine secretion from non-senescent cells (Figure 2). Collectively, these results suggest that LAV-BPIFB4 mitigates SASP induction triggered by ETP in GBM cells.

### 3.3. Recombinant Human LAV-BPIFB4 Sensitized U87-MG Cells to TMZ Treatment

Recent evidence suggests that tumor cells acquiring the ability to senesce become more resistant to long-term treatment with chemotherapeutic agents. Because a way to resist chemotherapy is through multi-drug-resistant P-glycoprotein (Pgp) upregulation, which acts both as a drug efflux pump and as an inhibitor of apoptosis [42], we speculated that ETP-treated U87-MG cells may show high surface levels of MDR after the acquisition of drug-induced senescence phenotype.

**Figure 3:**
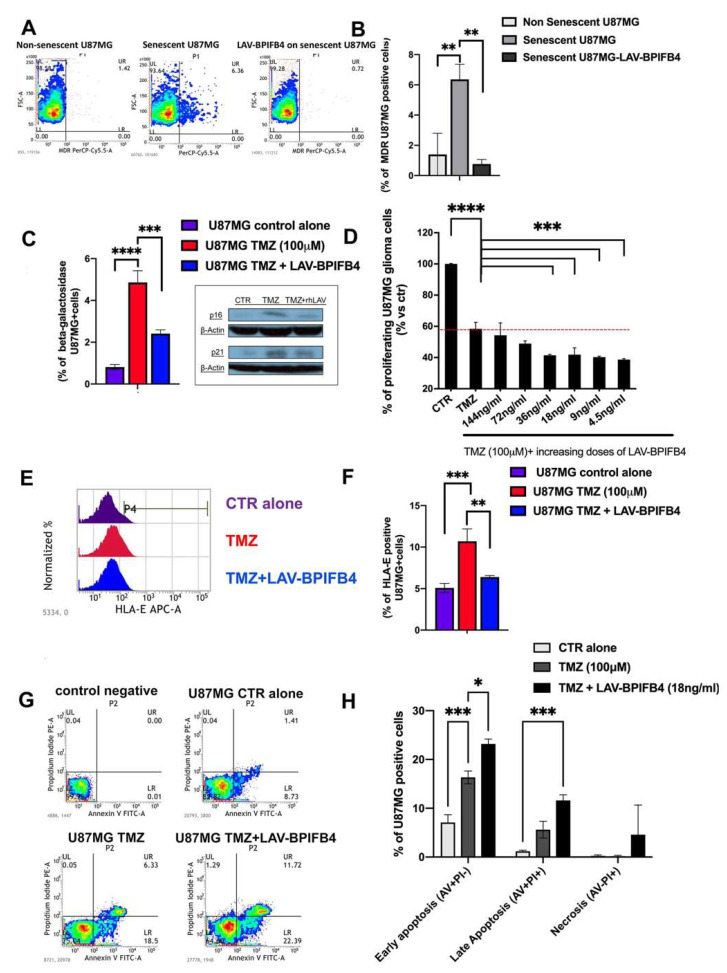
Drug sensibilization in senescent U87-MG glioblastoma cell line. (**A**) Representative FACS dot plots showing the percentages of multidrug resistance protein (MDR)-positive U87-MG cells in different conditions as indicated. After inducing senescence with etoposide, the percentage of MDR-positive cells rose to 6.36%, and the 48 h treatment with rhLAV-BPIFB4 reduced this value to 0.72%. (**B**) Bar graph reporting the mean percentage values ± SD of MDR+ viable cells from 3 independent experiments. (**C**) Cytofluorometric analysis of Spider-β galactosidase in the U87-MG glioma cell line after 24 h treatment with TMZ (100 μM) and 5 days resting in the absence or presence with rhLAV-BPIFB4 for the last 48 h. The right panel reports the Western blot analysis of p16 and p21 senescence-related proteins in the same experimental condition. β-Actin was used as a control for quantitation of the sample protein. (**D**) BrdU proliferation assay on the U87-MG cell line after treatment with temozolomide (96 h, 100 μM). The LAV-BPIFB4 co-treatment induced a higher sensibilization to the drug in a dose–response manner. (**E**) Cytofluorimetric analysis of the HLA-E expression. The panel on left side shows a representative, of three independent experiments, histogram profile of HLA-E staining on U87-MG cells’ surface in the control and TMZ-treated cells with or without rhLAV-BPIFB4 for the last 48 h of the 5 days resting. (**F**) The right panel is a bar graph of percentage positive cells. The results are representative of 3 independent experiments expressed as the mean ± SD. (**G**,**H**) Induction of apoptosis measured by Annexin V and propidium iodide (PI) double-staining through flow cytometry in the U87-MG glioma cell line after 24 h of treatment with TMZ (100 μM) and 5 days resting in the absence or presence of rhLAV-BPIFB4 for the last 48 h. The left panel is a representative density plot of cytofluorometric analysis. Histograms on the right indicate total percentage of early (Annexin V-positive cells/PI-negative cells) and late apoptotic events (Annexin V/PI-double positive cells) as well as necrotic cells (Annexin V-negative cells/PI-positive cells). The results are representative of 3 independent experiments performed in duplicate and expressed as mean ± SD. Statistical analysis by two-way ANOVA with Tukey’s test for multiple comparison was conducted (* *p* < 0.01, ** *p* <0.001, *** *p* <0.0001, **** *p* < 0.00001).

To test our hypothesis, we performed FACS analysis of MDR protein levels on the surface of U87-MG parental and ETP-exposed senescent cells (Figure 3A). As expected, the results showed that ETP-treated senescent U87-MG cells did express higher level of MDR compared to parental U87-MG cells (Figure 3A,B). Of interest, the co-treatment of ETP-treated senescent U87-MG cells with LAV-BPIFB4 significantly reduced the percentage of MDR positive cells (Figure 3A,B). This opens the way for an emergent approach to enhance the activity of current and prospective anti-cancer therapeutics by reducing the senescence burden.

As we herein showed that LAV-BPIFB4 blunts the degree of the senescence of cancer cells, we speculated that LAV-BPIFB4-treated U87-MG cells may also show high sensitivity to TMZ, the first-choice chemotherapeutic agent in GBM. To test our idea, first we proved the ability of LAV-BPIFB4 to interfere with the TMZ-induced senescence. Indeed, when we exposed U87-MG to TMZ for 24 h and 5 additional days resting, we found a slight increase in senescence-associated β-galactosidase (SA-β-gal) positive cells (Figure 3C, histograms) characterized by a higher level of p21 and p16 senescence-related markers (Figure 3C, WB analysis). Of note, when U87-MG cells were exposed to LAV-BPIFB4 during the last 48 h after TMZ treatment, the population of SA-β-gal-positive cells decreased. Likewise, LAV-BPIFB4 treatment induced a significative reduction of the cell cycle regulator p16 and p21, known to be essential for maintenance of the senescent cell cycle arrest [43]. As expected, the senotherapeutic effect of the LAV-BPIFB4 was also supported by the reduced HLA-E expression (Figure 3E,F) [41].

Thus, we moved to analyze the effects of combinations of TMZ and LAV-BPIFB4 in U87-MG cells in a proliferation assay. For TMZ, the fixed concentration of 100 μM was chosen. At this concentration (100 μM), the percentage of proliferating cells after 96 h was about 58 ± 3.1%. Interestingly, the co-treatment with lower doses of LAV-BPIFB4 (from 36 to 45 ng/mL) was more effective than TMZ alone in reducing proliferation of glioma cells (Figure 3D).

Together, these findings provide new insights on the potential role of LAV-BPIFB4 in inhibiting the effects of resistance to therapy. From a mechanistic point of view, LAV-BPIFB4 by reducing senescence (Figure 3C–F) might restore the ability of U87-MG cells to reenter the cell cycle with the consequent strengthening of TMZ-induced toxicity. This was suggested by the significant enhancement of apoptosis (both at early and late events) when TMZ-treated U87-MG cells were also exposed to LAV-BPIFB4 compared to TMZ-treated cells in which senescence and TMZ-resistance was shown (Figure 3G,H).

### 3.4. LAV-BPIFB4 Reversed the Senescent Phenotype of T cells from GBM Patients

The SASP has both paracrine and autocrine effects in maintaining senescence and spreading senescence to neighboring cells. The immune cells can sense the *low-grade* inflammatory background of the SASP by becoming aberrantly senescent themselves (immunosenescence). This impairment of the immune system results in compromised immune surveillance that is vital for tumor progression and metastasis.

To examine whether the LAV-BPIFB4 was also effective in reducing immunosenescence, we took advantage of the analysis of primary PBMCs isolated from GBM cancer patients including *low-grade* II–III (*N* = 6), and *high-grade* (*N* = 7) patients.

**Figure 4:**
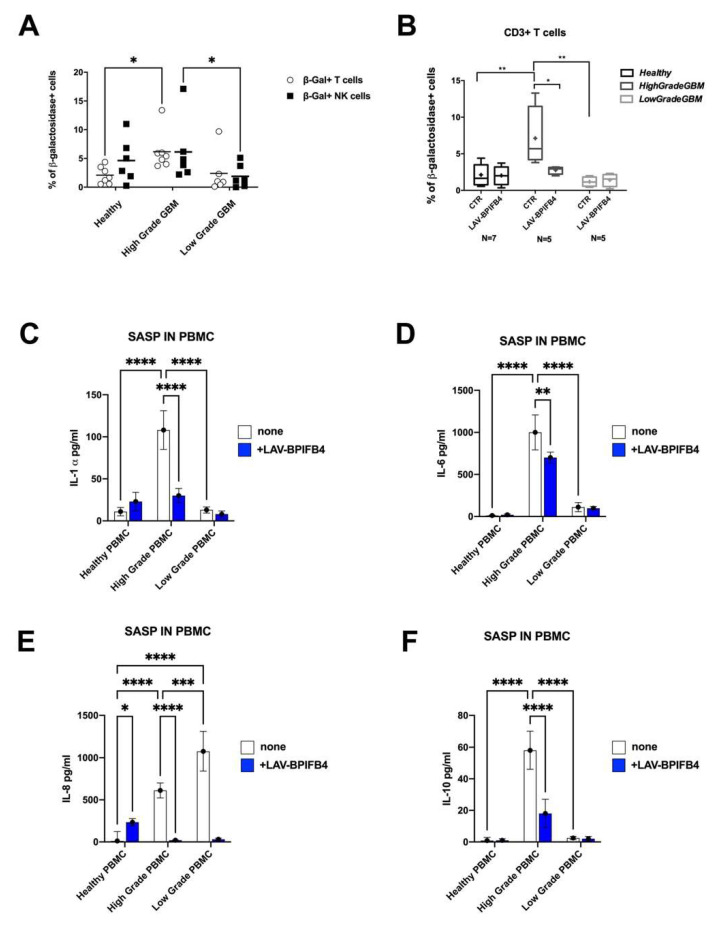
Immune senescence profile of PBMCs from GBM patients. (**A**) Results of the FACS analysis of Spider-β galactosidase in CD3+ T cells and CD3-CD56+ NK cells compartment among total PBMCs from GBM patients (*low* and *high* grade) and healthy control, for comparison. (**B**) Analysis of the effects of 48 h LAV-BPIFB4 treatment (18 ng/mL) on the percentage of Spider-βGal+ CD3+ T cells among total PBMCs from healthy donors, *high-* and *low-*grade GBM patients. *High-*grade GBM patients had more senescent CD3+ T cells then healthy or *low*-grade patients. The treatment with rhLAV-BPIFB4 could restore the profile of peripheral immune cells. Statistical analysis by two-way ANOVA with Tukey’s test for multiple comparison was conducted. (* *p* < 0.01, ** *p* < 0.001, *** *p* < 0.0001, **** *p* < 0.00001). (**C**–**F**) Secretory profile of PBMC from GBM patients (*low* and *high* grade) and healthy control after 48 h treatment with 18 ng/mL of rhLAV-BPIFB4 as detected by multiplex ELISA of the cell medium. Statistical analysis by two-way ANOVA with Tukey’s test for multiple comparison was conducted (* *p* < 0.01, ** *p* < 0.001, *** *p* < 0.0001, **** *p* < 0.00001).

Through FACS analysis, SA-β-gal substrate was used to identify both senescent CD3+ T cells and CD56^+^CD3^−^ NK cells in freshly isolated PBMCs. As a control, healthy donors (*N* = 7) were also analyzed. As depicted in Figure 4A, we monitored an increase in SA-beta Gal activity in peripheral CD3+ T cells of *high-grade* patients. Concerning the percentage of senescent NK cells, a statistical increase was found only when we compared *high-grade* vs. *low-grade* patients, suggesting its relationship with the pathology severity. Notably, the 48 h in vitro treatment of patients’ PBMCs with rhLAV-BPIFB4 resulted in a significant decrease in the senescent pool of peripheral T lymphocytes compared to untreated patients’ PBMCs (Figure 4B).

Of note, concerning the NK cell compartment, LAV-BPIFB4 was able to rescue the expression of the natural cytotoxicity receptor (NCR) NKp44 upon co-stimulation with interleukin (IL)-2. Indeed, in our cohort, the surface levels of NKp44, the only “inducible” receptor instrumental to immunosurveillance, were found downregulated in *high-grade* patients (2.01% ± 0.4 of positive NK cells) compared to healthy donors (3.66 ± 0.3% of positive NK cells) and *low-grade* patients (4.02 ± 1.2% of positive NK cells) (Supplementary Materials Figure S2). Notably, the percentage of NKp44+ NK cells from *high-grade* patients reached 3.89 ± 0.2% after LAV-BPIFB4 treatment (Supplementary Materials Figure S2).

Further, PBMC from *high-grade* GBM patients secreted higher levels of the SASP factors (i.e., IL-1α, IL-6, IL-8, and IL-10) than healthy and *low-grade* GBM patients (Figure 4C–F). As expected, LAV-BPIFB4 selectively suppressed the cytokine release from *high-grade* PBMC, indicative of a complete reversal of their cellular senescence phenotype (Figure 4C–F).

Contrary to the lymphoid compartment (Figure 4), CD11b^+^ cells, mainly monocytes, neutrophils, granulocytes, and macrophages, displayed a higher SA-β-gal activity in healthy PBMCs compared to *high-grade* and *low-grade* GBM patients (Supplementary Materials Figure S3A). This is probably because for immune cells with an intense lysosomal activity, SA-β-gal+ is not always indicative of senescence but marks cells with a normal cellular function compromised in cancer patients [44].

Then we asked if GBM immunosenescence may reflect an imbalance in the peripheral monocyte subsets. In PBMC from both healthy and *high-grade* and *low-grade* GBM patients, a higher frequency of inflammatory CD14+CD206+ M1-like monocytes was found compared to suppressive CD14+CD163+ M2-like monocytes (Supplementary Materials Figure S3C). Once again, no significant changes of monocyte phenotype were reported following LAV-BPIFB4 treatment (Supplementary Materials Figure S3D,E), suggesting its peculiar senotherapeutic activity on T-cell immunesenescence in cancer patients.

In conclusion, the ability of LAV-BPIFB4 to enhance health/longevity and cellular homeostasis might be useful in the future to control the senescence process occurring in the cancer microenvironment.

## 4. Conclusions and Perspectives

In the current study we found that LAV-BPIFB4, the recombinant product of the longevity-associated variant encoded by a four-SNP haplotype of the BPIFB4 gene, was associated to healthy aging and cellular homeostasis in several in vitro and in vivo contexts. It can reduce the senescence rate in both cultured U87-MG cell line and in the primary GBM patient-derived PBMCs. The senotherapeutic action of LAV-BPIFB4 was recently reported in aged-mice with the AAV-LAV-BPIFB4 gene transfer able to reduce the pool of peripheral immunosenescent cells in a CD38-NADase-dependent manner [45]. Previously, we also highlighted the importance of BPIFB4 levels to classify the health status of LLIs (by discriminating frail individuals vs. non-frail ones) [37]. In addition, by transferring healthy characteristics of longevity, LAV-BPIFB4 gene therapy was found to be effective in halting the progression of age-related conditions such as diabetic cardiomyopathy and atherosclerosis [34,35]. Finally, the LAV-BPIFB4 haplotype correlated with reduced frailty in a cohort of 237 elderly subjects and AAV-LAV transfer reduced frailty progression in old mice [36].

Here, we disclosed a new anti-senescence effect of LAV-BPIFB4 useful for GBM tumor control. Indeed, LAV-BPIFB4 treatment downregulates senescence both in cancer cells in vitro and in T lymphocytes from GBM patients, a key event which may support a dual activity of LAV-BPIFB4 in the cancer microenvironment. We demonstrated that LAV-BPIFB4 can specifically counterbalance the therapy-induced senescence (Figure 1 and Figure 3), a state which is responsible for a chronic secretory phenotype that is generally considered pro-tumorigenic and promigratory, especially in GBM residual disease [46].

We reported that LAV-BPIFB4 treatment reduces selectively IL-6 and IL-8 (Figure 2), from U87-MG cells, two cytokines known to maintain glioma stemness and create a supportive niche for GBM recurrence [47]. Consequently, LAV-BPIFB4 downregulated the surface expression of the non-classical MHC molecule HLA-E on the senescent U87-MG cells (Figure 1C and Figure 3E), recently reported to be induced by SASP-related pro-inflammatory cytokines reducing GBM immunogenicity [41]. In a putative treatment in vivo, this effect along with the NKp44 upregulation on the peripheral NK cells (Supplementary Materials Figure S2), might be responsible for the proper elimination of the senescent cells thus potentiating the immune response against the tumor. As a supposed mechanism, LAV-BPIFB4 may act at transcriptional level, as the regulation of the NKp44 gene contains many binding sites for the transcription factors AP-1, Oct-1, HNF-4, and Pax-4 [48], common clock-controlled genes in mammals [49]. Among the plethora of the immunosuppressive mechanisms, T-cell senescence and exhaustion in response to persistent cancer cell stimulation represent another major driver of dysfunctional immune responses of the GBM and of many other cancers [27,50]. The occurrence of this event is accentuated in elderly patients having a higher number of senescent T cells and thymic shrinkage; this might explain why the LAV haplotype in the BPIFB4 gene, selected by evolutionary forces [38] and able to counteract the age-related decline, was also effective in reversing the T-cell senescence in PBMCs from GBM patients (Figure 4). No changes in peripheral monocytes’ phenotype were reported in our experimental setting (Supplementary Materials Figure S3). From our perspective, the analysis of the mono-macrophage tumor infiltrating cells, which have been described to be more impaired than the peripheral counterpart [50], will be a more suitable cell model to test the polarizing effect of LAV-BPIFB4.

As regards the cancer cell compartment, we tested the LAV-BPIFB4 effect against the senescence response upon both ETP or TMZ treatment (Figure 1 and Figure 3, respectively). In our opinion, the downregulation of MDR level by LAV-BPIFB4 on the surface of ETP-treated senescent cells (Figure 3A) may be a general mechanism valid also for explaining the better response to TMZ (Figure 3C–F). Indeed, the inhibition and control of the MDR complex have been widely found in correlating with an enhanced GBM sensitivity to temozolomide [24,51]. Furthermore, in TMZ-exposed glioma cells, the pre-treatment with LAV-BPIFB4, by reducing senescence (Figure 3C–F) and probably by restoring the ability of these cells to reenter the cell cycle (as preliminarily documented in Hek-293 transfected with LAV-BPIFB4 isoforms, *unpublished data*) may cause the TMZ-induced toxicity, as suggested by the higher apoptotic rate induced by LAV-BPIFB4 co-treatment (Figure 3G,H). In this context, Granada A. et al. recently reported the unexpected effect of proliferation status in regulating responses to cisplatin and suggest that slowly proliferating cells within tumors, like those reentering the cell cycle, may be acutely vulnerable to chemotherapy [52]. In the same way, the response to neoadjuvant chemotherapy has been strongly associated with proliferation of ER^+^/HER2^−^ breast cancers [53].

Furthermore, the capability of LAV-BPIFB4 to delay aging may be an adjunct value to its putative anti-GBM action. Indeed, age is a negative prognostic factor for GBM stage, due to the fact of senescence increase, which causes the enhancement of the tumor-supportive senescence state in brain parenchyma. Further we must consider that the systemic administration of cancer-therapy often generates a pool of senescent cells also in non-malignant areas, which may cause the early onset and progression of chronic diseases such as cardiovascular, fibrotic, and neurodegenerative diseases, and which are normally observed at an advanced age [54,55]. Thus, new strategies based on LAV-BPIFB4 administration may be useful to limit the detrimental effects of therapy-induced senescent cells by rescuing tissue impairment in cancer patients.

Another goal of the cancer therapy is to consider several personal factors before selecting a cancer treatment. This helps predict an individual’s response to therapy and find the most effective treatment. Compared to WT, homozygous LAV carriers have higher circulating BPIFB4 levels and a better response to counteract age-induced impairment. These led us to speculate that cancer subjects carrying the LAV-BPIFB4 genotype might display reduced chemotherapy resistance and lower adverse reactions to cancer treatment as well as a favorable immune profile. If the overall hypothesis is correct, BPIFB4 polymorphism may be characterized as an independent prognostic factor of response to cancer treatment.

In summary, even though the related mechanisms of such effects need to be deeply dissected, in the future, treatment with LAV-BPIFB4 as well as other senotherapeutics, might be a valuable strategy to restore therapy-sensitivity and a proper immune surveillance.

## Data Availability

The authors confirm that the data supporting the findings of this study are available within the article and its supplementary materials. Raw data are available from the corresponding authors, [E.C and A.A.P], upon reasonable request.

## Supplementary Materials

The following supporting information can be downloaded at: https://www.mdpi.com/article/10.3390/cells11020294/s1, Figure S1: Effects of rhLAV-BPIFB4 on growth and cellular integrity of human U87-MG glioblastoma cell line. Figure S2: Effects of rhLAV-BPIFB4 on the NKp44 expression on primary NK cells from GBM patients. Figure S3: Analysis of the monocyte profile in PBMC of healthy donor and GBM patients.

## References

[1] Quail D.F., Joyce J.A. (2017). The microenvironmental landscape of brain tumors. Cancer Cell.

[2] Stupp R., Mason W.P., van den Bent M.J., Weller M., Fisher B., Taphoorn M.J., Belanger K., Brandes A.A., Marosi C., Bogdahn U. (2005). Radiotherapy plus concomitant and adjuvant temozolomide for glioblastoma. N. Engl. J. Med..

[3] Weller M., van den Bent M., Tonn J.C., Stupp R., Preusser M., Cohen-Jonathan-Moyal E., Henriksson R., Le Rhun E., Balana C., Chinot O. (2017). European Association for Neuro-Oncology (EANO) guideline on the diagnosis and treatment of adult astrocytic and oligodendroglial gliomas. Lancet Oncol..

[4] Haanen J.B.A.G., Carbonnel F., Robert C., Kerr K.M., Peters S., Larkin J., Jordan K., ESMO Guidelines Committee (2017). Management of toxicities from immunotherapy: ESMO Clinical Practice Guidelines for diagnosis, treatment and follow-up. Ann. Oncol..

[5] Sonabend A.M., Carminucci A.S., Amendolara B., Bansal M., Leung R., Lei L., Realubit R., Li H., Karan C., Yun J. (2014). Convection-enhanced delivery of etoposide is effective against murine proneural glioblastoma. Neuro Oncol..

[6] Smith S.J., Tyler B.M., Gould T., Veal G.J., Gorelick N., Rowlinson J., Serra R., Ritchie A., Berry P., Otto A. (2019). Overall Survival in Malignant Glioma Is Significantly Prolonged by Neurosurgical Delivery of Etoposide and Temozolomide from a Thermo-Responsive Biodegradable Paste. Clin. Cancer Res..

[7] Brenner E., Schörg B.F., Ahmetlić F., Wieder T., Hilke F.J., Simon N., Schroeder C., Demidov G., Riedel T., Fehrenbacher B. (2020). Cancer immune control needs senescence induction by interferon-dependent cell cycle regulator pathways in tumours. Nat. Commun..

[8] Xue W., Zender L., Miething C., Dickins R.A., Hernando E., Krizhanovsky V., Cordon-Cardo C., Lowe S.W. (2007). Senescence and tumour clearance is triggered by p53 restoration in murine liver carcinomas. Nature.

[9] Tamayo-Orrego L., Wu C.L., Bouchard N., Khedher A., Swikert S.M., Remke M., Skowron P., Taylor M.D., Charron F. (2016). Evasion of Cell Senescence Leads to Medulloblastoma Progression. Cell Rep..

[10] Roninson I.B. (2003). Tumor cell senescence in cancer treatment. Cancer Res..

[11] Eggert T., Wolter K., Ji J., Ma C., Yevsa T., Klotz S., Medina-Echeverz J., Longerich T., Forgues M., Reisinger F. (2016). Distinct Functions of Senescence-Associated Immune Responses in Liver Tumor Surveillance and Tumor Progression. Cancer Cell.

[12] Korkaya H., Kim G.I., Davis A., Malik F., Henry N.L., Ithimakin S., Quraishi A.A., Tawakkol N., D’Angelo R., Paulson A.K. (2012). Activation of an IL6 inflammatory loop mediates trastuzumab resistance in HER^2+^ breast cancer by expanding the cancer stem cell population. Mol. Cell.

[13] Chen X., Mitsutake N., LaPerle K., Akeno N., Zanzonico P., Longo V.A., Mitsutake S., Kimura E.T., Geiger H., Santos E. (2009). Endogenous expression of Hras(G12V) induces developmental defects and neoplasms with copy number imbalances of the oncogene. Proc. Natl. Acad. Sci. USA.

[14] Ritschka B., Storer M., Mas A., Heinzmann F., Ortells M.C., Morton J.P., Sansom O.J., Zender L., Keyes W.M. (2017). The senescence-associated secretory phenotype induces cellular plasticity and tissue regeneration. Genes Dev..

[15] Chiche A., Le Roux I., von Joest M., Sakai H., Aguín S.B., Cazin C., Salam R., Fiette L., Alegria O., Flamant P. (2017). Injury-Induced Senescence Enables In Vivo Reprogramming in Skeletal Muscle. Cell Stem Cell.

[16] Aasland D., Götzinger L., Hauck L., Berte N., Meyer J., Effenberger M., Schneider S., Reuber E.E., Roos W.P., Tomicic M.T. (2019). Temozolomide Induces Senescence and Repression of DNA Repair Pathways in Glioblastoma Cells via Activation of ATR–CHK1, p21, and NF-kB. Cancer Res..

[17] Mehta A., Awah C.U., Sonabend A.M. (2018). Topoisomerase II Poisons for Glioblastoma; Existing Challenges and Opportunities to Personalize Therapy. Front. Neurol..

[18] Kurz D.J., Decary S., Hong Y., Erusalimsky J.D. (2000). Senescence-associated (beta)-galactosidase reflects an increase in lysosomal mass during replicative ageing of human endothelial cells. J. Cell Sci..

[19] Kosar M., Bartkova J., Hubackova S., Hodny Z., Lukas J., Bartek J. (2011). Senescence-Associated heterochromatin foci are dispensable for cellular senescence, occur in a cell type- and insult-dependent manner and follow expression of p16(ink4a). Cell Cycle.

[20] d’Adda di Fagagna F. (2008). Living on a break: Cellular senescence as a DNA-damage response. Nat. Rev. Cancer.

[21] Campisi J., d’Adda di Fagagna F. (2007). Cellular senescence: When bad things happen to good cells. Nat. Rev. Mol. Cell Biol..

[22] Saleh T., Tyutynuk-Massey L., Cudjoe Jr E.K., Idowu M.O., Landry J.W., Gewirtz D.A. (2018). Non-Cell Autonomous Effects of the Senescence-Associated Secretory Phenotype in Cancer. Front. Oncol..

[23] Johannessen T.C., Bjerkvig R. (2012). Molecular mechanisms of temozolomide resistance in glioblastoma multiforme. Expert Rev. Anticancer Ther..

[24] Tivnan A., Zakaria Z., O’Leary C., Kögel D., Pokorny J.L., Sarkaria J.N., Prehn J.H. (2015). Inhibition of multidrug resistance protein 1 (MRP1) improves chemotherapy drug response in primary and recurrent glioblastoma multiforme. Front. Neurosci..

[25] Bao S., Wu Q., Sathornsumetee S., Hao Y., Li Z., Hjelmeland A.B., Shi Q., McLendon R.E., Bigner D.D., Rich J.N. (2006). Stem cell-like glioma cells promote tumor angiogenesis through vascular endothelial growth factor. Cancer Res..

[26] Wei J., Barr J., Kong L.Y., Wang Y., Wu A., Sharma A.K., Gumin J., Henry V., Colman H., Priebe W. (2010). Glioblastoma cancer-initiating cells inhibit T-cell proliferation and effector responses by the signal transducers and activators of transcription 3 pathway. Mol. Cancer Ther..

[27] Woroniecka K.I., Rhodin K.E., Chongsathidkiet P., Keith K.A., Fecci P.E. (2018). T-Cell Dysfunction in Glioblastoma: Applying a New Framework. Clin. Cancer Res..

[28] Campisi J. (2013). Aging, cellular senescence, and cancer. Annu. Rev. Physiol..

[29] Ostrom Q.T., Gittleman H., Xu J., Kromer C., Wolinsky Y., Kruchko C., Barnholtz-Sloan J.S. (2016). CBTRUS Statistical Report: Primary Brain and Other Central Nervous System Tumors Diagnosed in the United States in 2009–2013. Neuro. Oncol..

[30] Louis D.N., Perry A., Reifenberger G., von Deimling A., Figarella-Branger D., Cavenee W.K., Ohgaki H., Wiestler O.D., Kleihues P., Ellison D.W. (2016). The 2016 World Health Organization Classification of Tumors of the Central Nervous System: A summary. Acta Neuropathol..

[31] Walford R.L. (1964). The Immunologic Theory of Aging. Gerontologist.

[32] Ciaglia E., Lopardo V., Montella F., Sellitto C., Manzo V., De Bellis E., Iannaccone T., Franci G., Zannella C., Pagliano P. (2021). BPIFB4 Circulating Levels and Its Prognostic Relevance in COVID-19. J. Gerontol. A Biol. Sci. Med. Sci..

[33] Di Pardo A., Ciaglia E., Cattaneo M., Maciag A., Montella F., Lopardo V., Ferrario A., Villa F., Madonna M., Amico E. (2020). The longevity-associated variant of BPIFB4 improves a CXCR4-mediated striatum–microglia crosstalk preventing disease progression in a mouse model of Huntington’s disease. Cell Death Dis..

[34] Dang Z., Avolio E., Thomas A.C., Faulkner A., Beltrami A.P., Cervellin C., Carrizzo A., Maciag A., Gu Y., Ciaglia E. (2020). Transfer of a human gene variant associated with exceptional longevity improves cardiac function in obese type 2 diabetic mice through induction of the SDF-1/CXCR4 signalling pathway. Eur. J. Heart Fail..

[35] Puca A.A., Carrizzo A., Spinelli C., Damato A., Ambrosio M., Villa F., Ferrario A., Maciag A., Fornai F., Lenzi P. (2020). Single systemic transfer of a human gene associated with exceptional longevity halts the progression of atherosclerosis and inflammation in ApoE knockout mice through a CXCR4-mediated mechanism. Eur. Heart J..

[36] Malavolta M., Dato S., Villa F., De Rango F., Iannone F., Ferrario A., Maciag A., Ciaglia E., D’Amato A., Carrizzo A. (2019). LAV-BPIFB4 associates with reduced frailty in humans and its transfer prevents frailty progression in old mice. Aging.

[37] Villa F., Malovini A., Carrizzo A., Spinelli C.C., Ferrario A., Maciąg A., Madonna M., Bellazzi R., Milanesi L., Vecchione C. (2015). Serum BPIFB4 levels classify health status in long-living individuals. Immun. Ageing..

[38] Dossena M., Ferrario A., Lopardo V., Ciaglia E., Puca A.A. (2020). New Insights for BPIFB4 in Cardiovascular Therapy. Int. J. Mol. Sci..

[39] Ciaglia E., Montella F., Maciag A., Scala P., Ferrario A., Banco C., Carrizzo A., Spinelli C.C., Cattaneo M., De Candia P. (2019). Longevity-Associated Variant of BPIFB4 Mitigates Monocyte-Mediated Acquired Immune Response. J. Gerontol. A Biol. Sci. Med. Sci..

[40] Tamamori-Adachi M., Koga A., Susa T., Fujii H., Tsuchiya M., Okinaga H., Hisaki H., Iizuka M., Kitajima S., Okazaki T. (2018). DNA damage response induced by Etoposide promotes steroidogenesis via GADD45A in cultured adrenal cells. Sci. Rep..

[41] Pereira B.I., Devine O.P., Vukmanovic-Stejic M., Chambers E.S., Subramanian P., Patel N., Virasami A., Sebire N.J., Kinsler V., Valdovinos A. (2019). Senescent cells evade immune clearance via HLA-E-mediated NK and CD8+ T cell inhibition. Nat. Commun..

[42] Ruth A.C., Roninson I.B. (2000). Effects of the multidrug transporter P-glycoprotein on cellular responses to ionizing radiation. Cancer Res..

[43] Stein G.H., Drullinger L.F., Soulard A., Dulić (1999). Differential roles for cyclin-dependent kinase inhibitors p21 and p16 in the mechanisms of senescence and differentiation in human fibroblasts. Mol. Cell Biol..

[44] Huang T., Rivera-Pérez J.A. (2014). Senescence-Associated β-galactosidase activity marks the visceral endoderm of mouse embryos but is not indicative of senescence. Genesis.

[45] Ciaglia E., Lopardo V., Montella F., Carrizzo A., Di Pietro P., Malavolta M., Giacconi R., Orlando F., Cattaneo M., Madeddu P. Transfer of the Longevity associated variant of BPIFB4 gene rejuvenates immune system and vasculature by a reduction of CD38+macrophages and NAD+ decline. Cell Death Dis..

[46] Putavet D.A., de Keizer P.L.J. (2021). Residual Disease in Glioma Recurrence: A Dangerous Liaison with Senescence. Cancers.

[47] Coppé J.P., Patil C.K., Rodier F., Sun Y., Muñoz D.P., Goldstein J., Nelson P.S., Desprez P.Y., Campisi J. (2008). Senescence-associated secretory phenotypes reveal cell-nonautonomous functions of oncogenic RAS and the p53 tumor suppressor. PLoS Biol..

[48] Ito K., Kawana M., Iwata T., Higai K. (2019). Transcriptional Regulation of the Natural Cytotoxicity Receptor NKp44 Gene in Human NK Cell Leukemia. J. Glycomics Lipidomics.

[49] Bozek K., Relógio A., Kielbasa S.M., Heine M., Dame C., Kramer A., Herzel H. (2009). Regulation of clock-controlled genes in mammals. PLoS ONE.

[50] Pearson J.R.D., Cuzzubbo S., McArthur S., Durrant L.G., Adhikaree J., Tinsley C.J., Pockley A.G., McArdle S.E.B. (2020). Immune Escape in Glioblastoma Multiforme and the Adaptation of Immunotherapies for Treatment. Front. Immunol..

[51] Munoz J.L., Rodriguez-Cruz V., Greco S.J., Nagula V., Scotto K.W., Rameshwar P. (2014). Temozolomide induces the production of epidermal growth factor to regulate MDR1 expression in glioblastoma cells. Mol. Cancer Ther..

[52] Granada A.E., Jiménez A., Stewart-Ornstein J., Blüthgen N., Reber S., Jambhekar A., Lahav G. (2020). The effects of proliferation status and cell cycle phase on the responses of single cells to chemotherapy. Mol. Biol. Cell.

[53] Stover D.G., Coloff J.L., Barry W.T., Brugge J.S., Winer E.P., Selfors L.M. (2016). The Role of Proliferation in Determining Response to Neoadjuvant Chemotherapy in Breast Cancer: A Gene Expression-Based Meta-Analysis. Clin. Cancer Res..

[54] Schosserer M., Grillari J., Breitenbach M. (2017). The Dual Role of Cellular Senescence in Developing Tumors and Their Response to Cancer Therapy. Front. Oncol..

[55] Demaria M., O’Leary M.N., Chang J., Shao L., Liu S., Alimirah F., Koenig K., Le C., Mitin N., Deal A.M. (2017). Cellular Senescence Promotes Adverse Effects of Chemotherapy and Cancer Relapse. Cancer Discov..

