# Supplementary material for: The Longevity-Associated Variant of BPIFB4 Reduces Senescence in Glioma Cells and in Patients’ Lymphocytes Favoring Chemotherapy Efficacy
Source: Cells. 2022 Jan 15;11(2):294. doi: 10.3390/cells11020294 (PMC8774353; doi:10.3390/cells11020294)
Supplement: Supplementary file 1 [file cells-11-00294-s001.zip › cells-1545403-SM-need to update.pdf]

Supplementary Figures

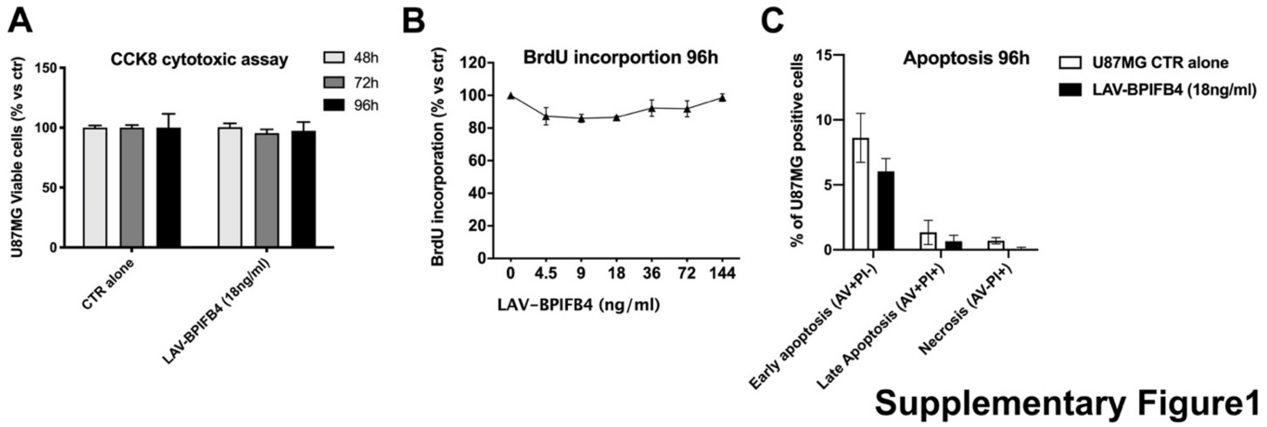

Supplementary Figure 1. Effects of rhLAV-BPIFB4 on growth and cellular integrity of human U87-MG glioblastoma cell line. **A** U87-MG cells were cultured in time course in the presence of the rhLAV-BPIFB4 before conducting the cytotoxic assay, using Cell Counting Kit-8 (*as better detailed in M&M section*). Results are expressed as means ± SD of 3 independent experiments performed in triplicate and reported as percentage of viable cells vs the untreated control. **B** U87-MG cells were cultured for 96h in the presence of the indicated concentrations (0–144 μM) of LAV-BPIFB4 before analysis of cell proliferation by BrdU incorporation assay. Results are expressed as means ± SD of 3 independent experiments performed in triplicate and reported as percentage vs the untreated control **C** Induction of apoptosis measured by annexin V and propidium iodide (PI) double staining through flow cytometry in 96h LAV-BPIFB4-treated U87-MG cells. Histograms indicate total percentage of early (Annexin V-positive cells/ PI-negative cells) and late apoptotic events (Annexin V/ PI-double positive cells) as well as necrotic cells (Annexin V-negative cells/ PI-positive cells). Results are representative of 3 independent experiments and expressed as mean ± SD.

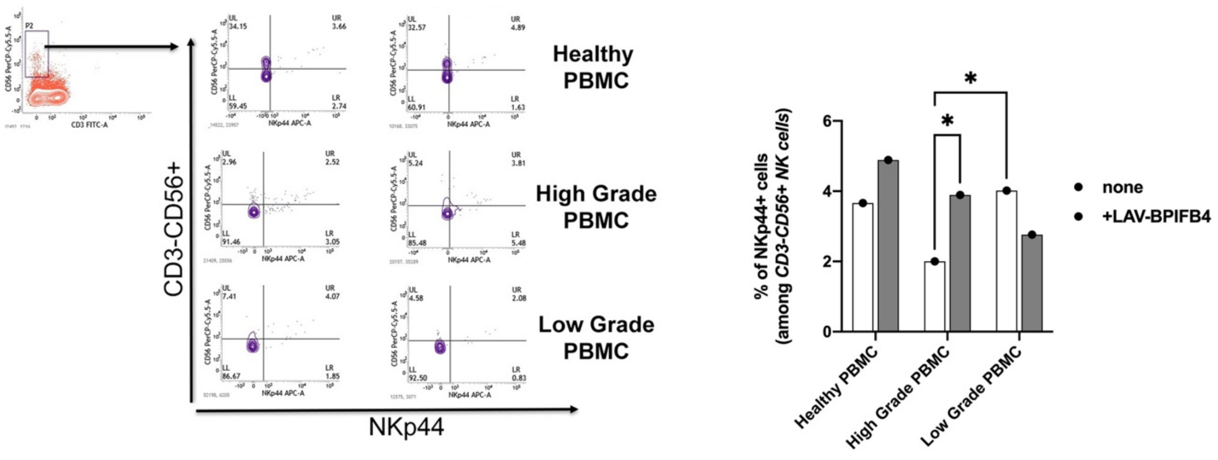

Supplementary Figure 2

Supplementary Figure 2. Effects of rhLAV-BPIFB4 on the NKp44 expression on primary NK cells from GBM patients. FACS analysis of NKp44 NCR receptor on CD3-/CD56+ positive NK cells among PBMC from healthy and *low and high grade* glioblastoma patients in basal condition and after 48h treatment with rhLAV-BPIFB4. The *left panel* shows the gating strategy and dot plot analysis from representative patients. The *right panel* is a bar graph showing the mean values percentage ± SD of NKp44+ positive cells based on at least 3 independent experiments and evaluated by ANOVA (*P < 0.05, compared with untreated cells).

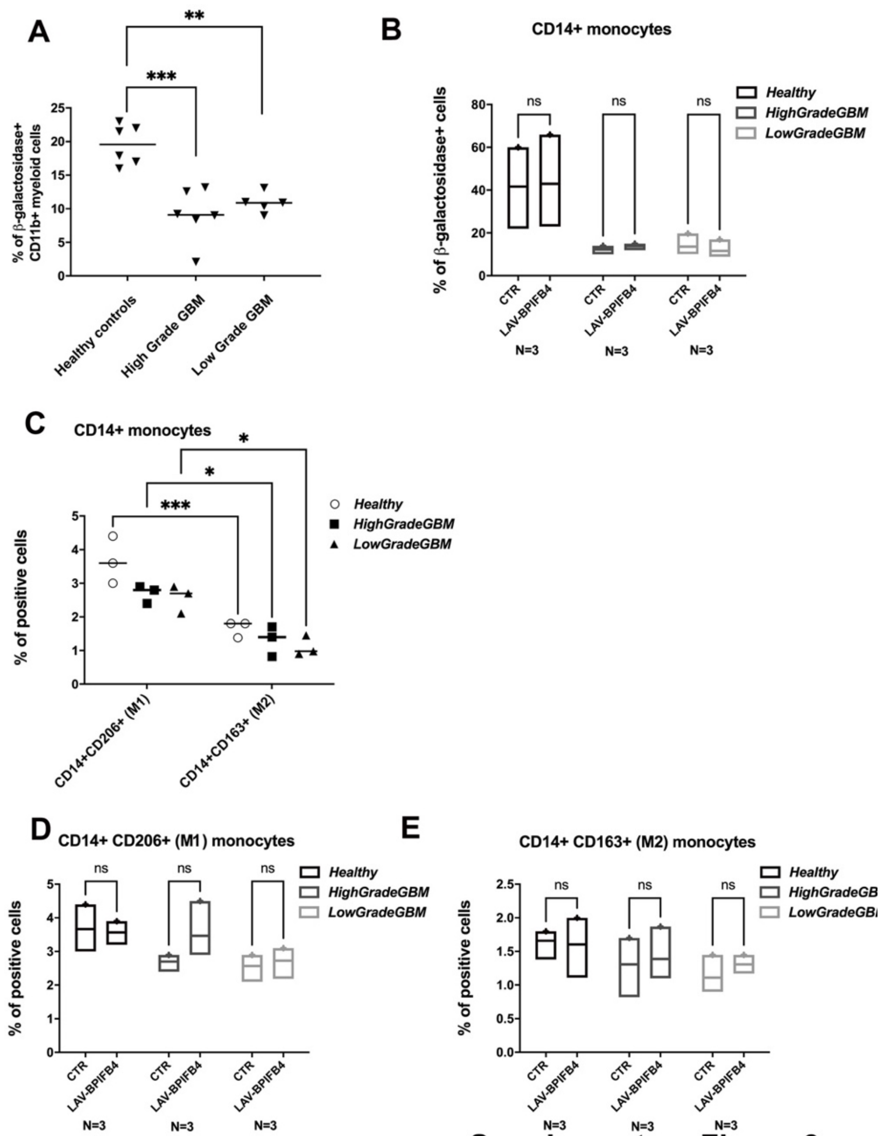

Supplementary Figure 3

Supplementary Figure 3. Analysis of the monocyte profile in PBMC of healthy donor and GBM patients. A Cytofluorimetric analysis of the immune senescence through Spider-β Galactosidase staining. The bar graph shows the percentage of Spider-β Galactosidase positive stained cells among CD11b+ myeloid cells and **B** in CD14+ positive cells in absence or presence of rhLAV-BPIFB4 at 18ng/ml for 48h. Healthy donor n=3, High grade GBM n=3 and low grade GBM n=3. **C** Cytofluorimetric analysis of M1/M2 balance in CD14+ positive cells from GBM patients (*low* and *high* grade) and healthy control, for comparison, using CD206 as M1 marker and CD163 as M2 marker. The graph represents the percentage of positive cells for the indicated markers. **D-E** Effects of rhLAV-BPIFB4 treatment on M1/M2 balance in patients and healthy donor's PBMC. Pairwise comparisons

statistically significant are indicated (*P<0.05, **P<0.01, ***P<0.001). Statistical evaluation was carried out by ordinary 2-way ANOVA corrected for Šídák's multiple comparisons test (GraphPad® Prism).
